# Supplementary material for: Ceramic nanowelding
Source: Nat Commun. 2018 Jan 8;9:96. doi: 10.1038/s41467-017-02590-1 (PMC5758820; doi:10.1038/s41467-017-02590-1)
Supplement: Supplementary file 1 — Supplementary Information [file 41467_2017_2590_MOESM1_ESM.pdf]

Supplementary Figures

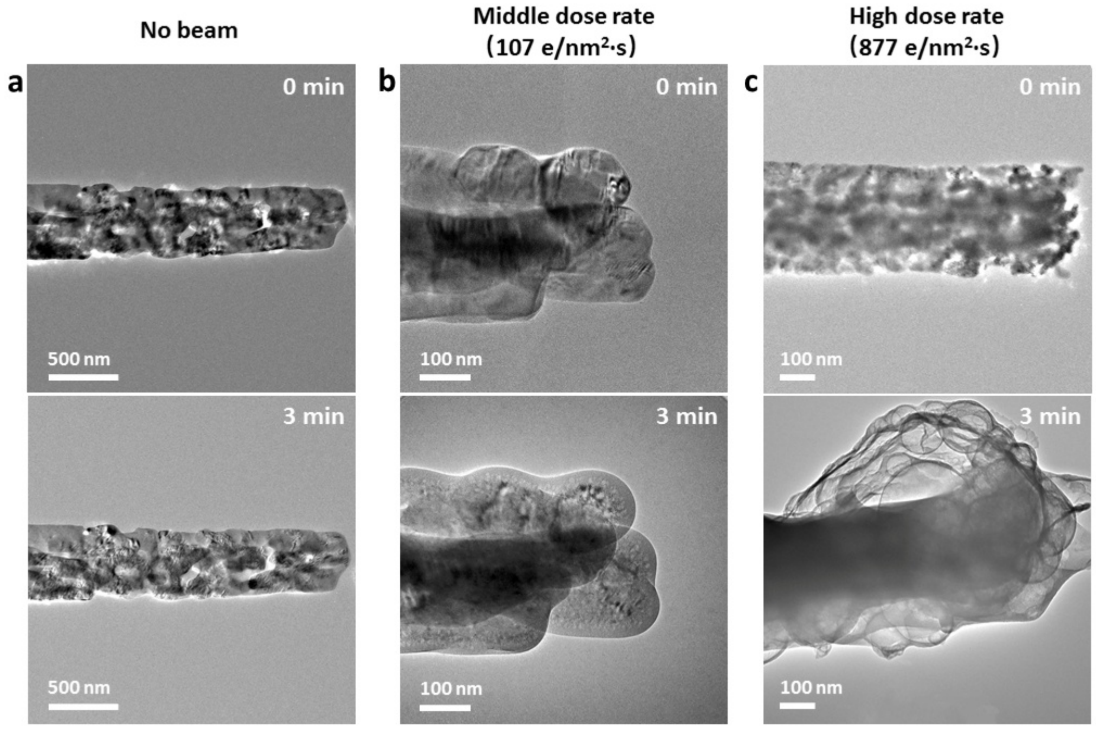

Supplementary Figure 1. Electron beam (e-beam) dose rate plays a significant role in the welding process. (a) When the e-beam is blank, no reaction occurred between the MgO and CO_2. (b) By increasing the e-beam dose rate to about 100 e/nm^2·s, MgO reacted with CO_2 with medium-speed. (c) When the e-beam irradiation dose rate reaches as high as 877 e/nm^2·s, a large amount of highly mobile bubbles emerged in the interior of the irradiated MgO nanowire.

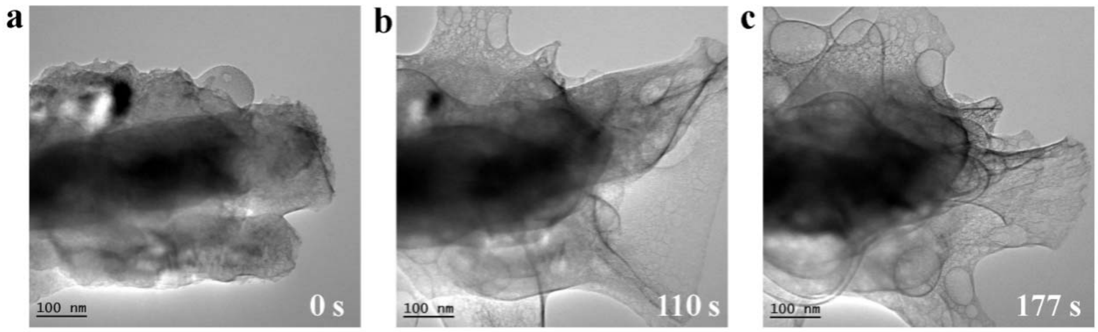

Supplementary Figure 2. The melting behavior of MgO under the e-beam irradiation in a CO_2 environment. **a**, 0 s, **b**, 110 s, **c**, 177 s.

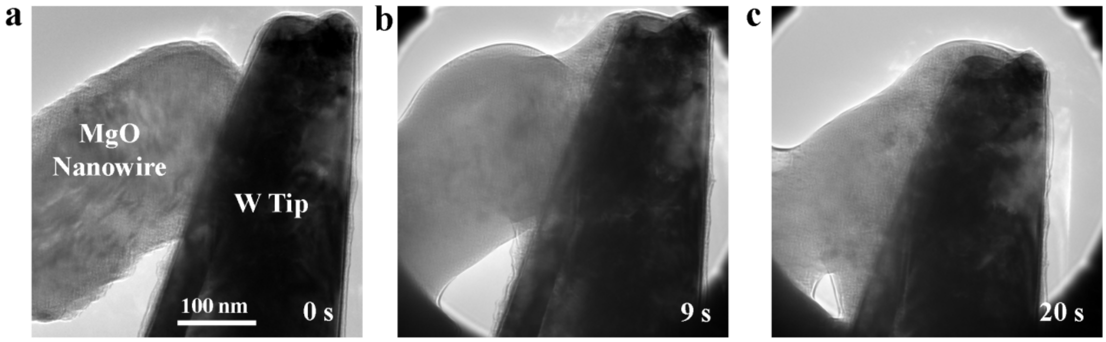

Supplementary Figure 3. The welding process of the MgO nanowire onto the W tip in a side-by-side mode. a, A MgO nanowire contacting the W tip. **b**, In the flow of CO_2 and under e-beam irradiation, the MgO nanowire was welded to the W tip. The morphology change after reaction for 9 s. **c**, The morphology change after reaction for 20 s.

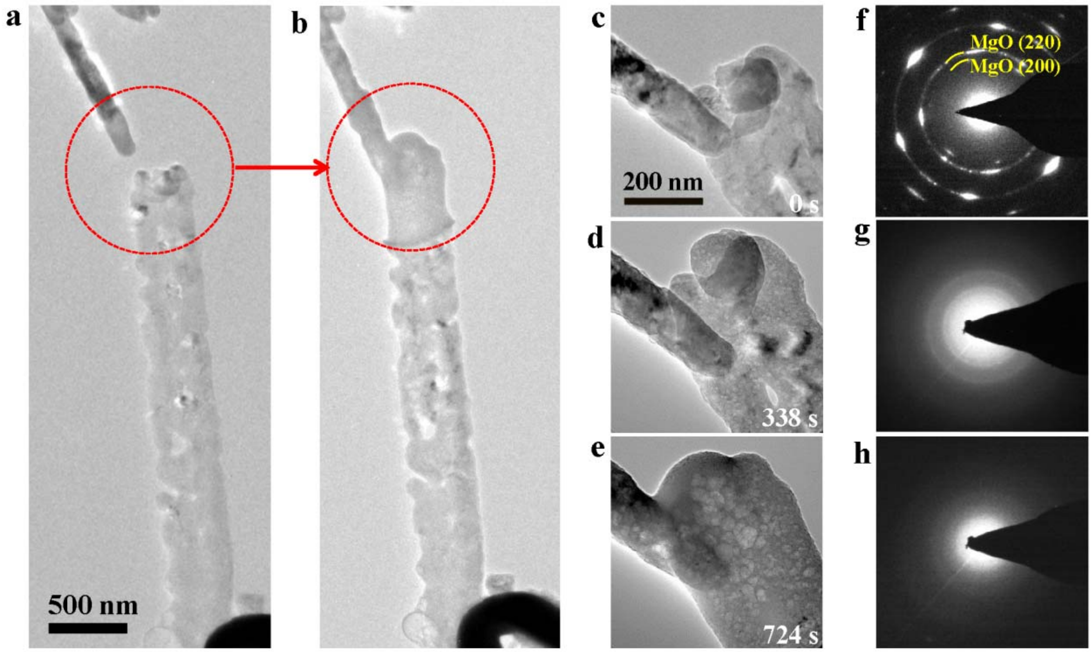

Supplementary Figure 4. A welding phenomenon occurred quickly under the e-beam irradiation in a CO_2 environment. a, Two MgO nanowires were approaching each other. Circles mark morphology changes of the same spot before (**a**) and after (**b**) welding. **c-e**, The morphology evolutions of the welded junction during the welding process. **f-h**, The electron diffraction patterns (EDPs) to **b-d**, respectively, showing the change from a polycrystalline MgO to an amorphous $MgCO_3$.

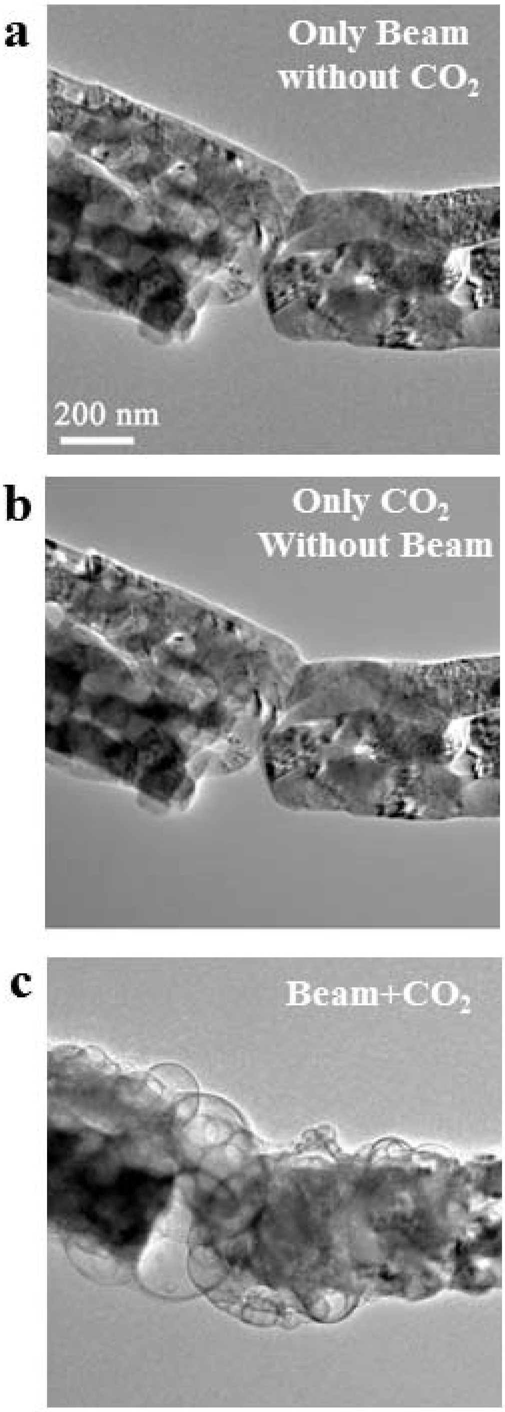

Supplementary Figure 5. Beam irradiation and CO_2 are two crucial factors to conduct a ceramic welding. The MgO morphology change under different conditions, including (**a**) only beam without CO_2. (**b**) only CO_2 without beam, and (**c**) beam combines with CO_2 respectively.

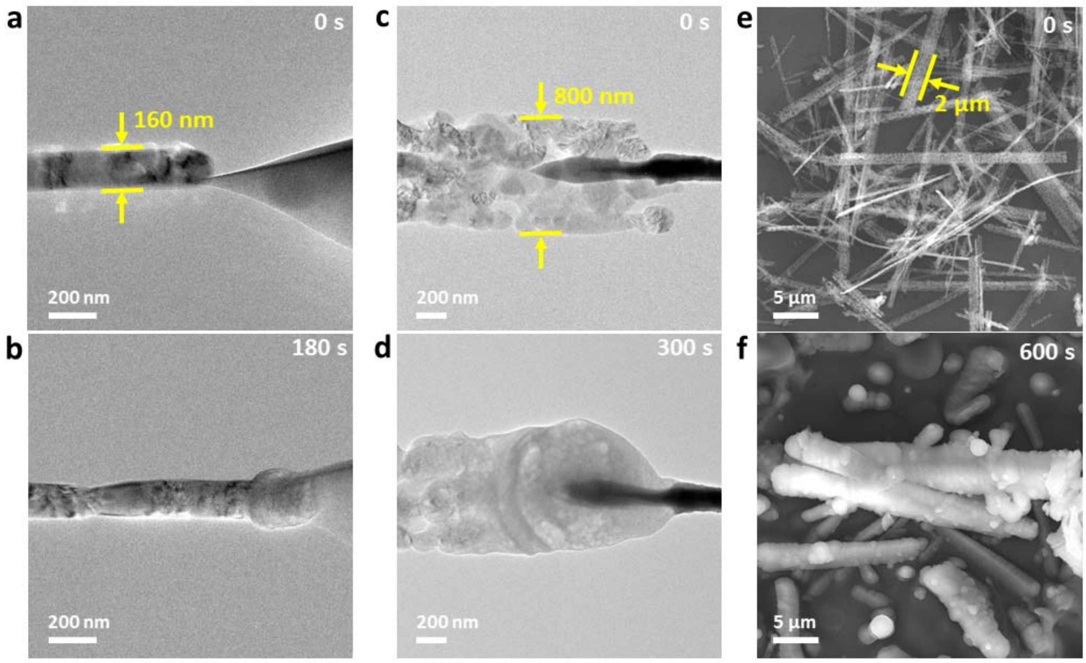

Supplementary Figure 6. Samples with different diameters are welded. (a) and (b), (c) and (d), (e) and (f) are MgO nanowires with different diameters before and after welding, respectively.

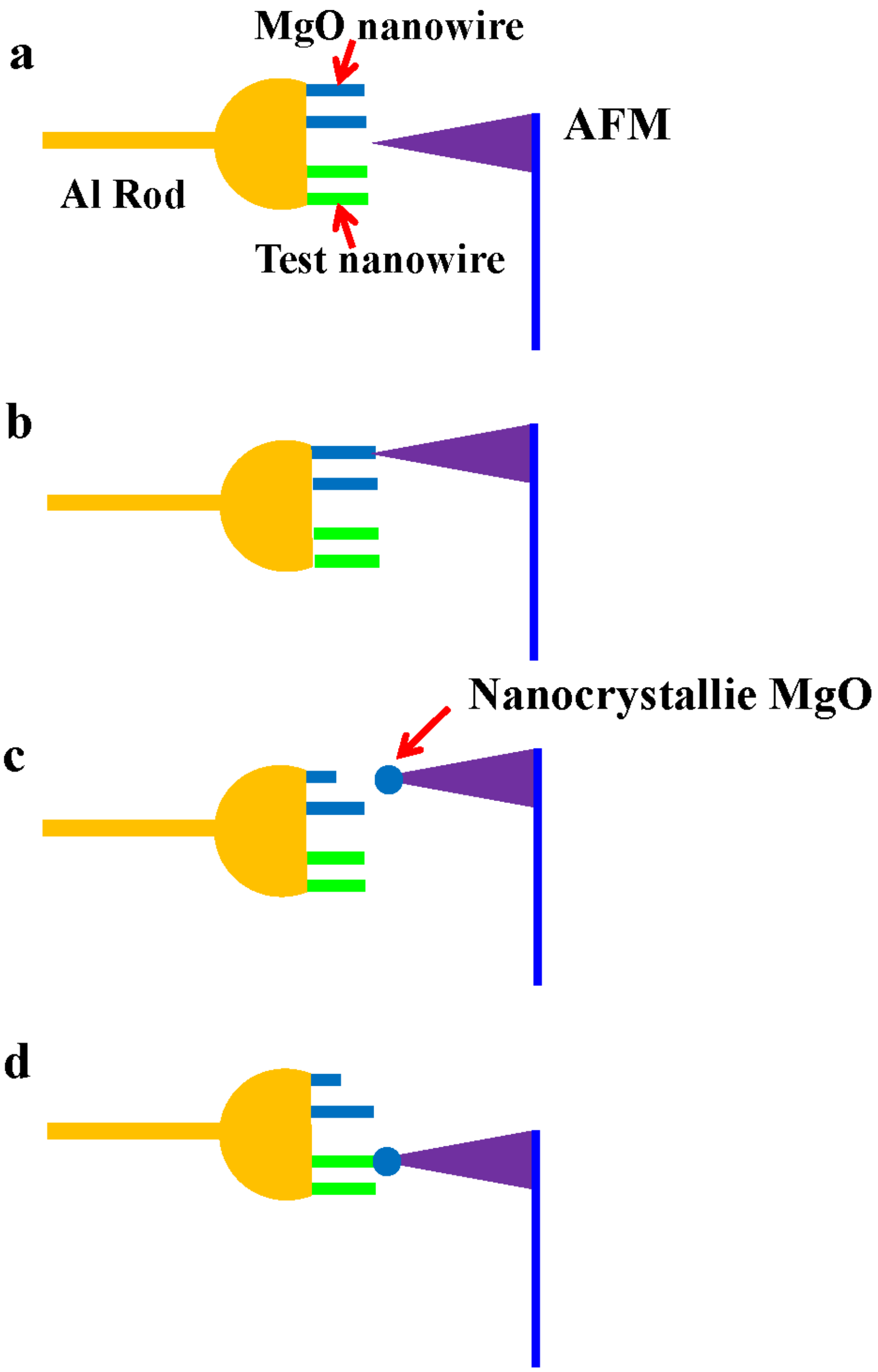

Supplementary Figure 7. The detailed welding steps for tensile testing of ceramic nanowires. **a**, Firstly, the MgO and the nanowires to be tested were mounted on either sides of the Al Rod with silver glue. **b**, The MgO nanowires were first attached to the AFM tip as the solder for welding. **c**, The Al rod was withdrawn, and some MgO nanocrystals were left on the AFM tip. **d**, Using the nanocrystalline MgO as a solder, the nanowires were welded to the AFM cantilever and ready for the tensile test.

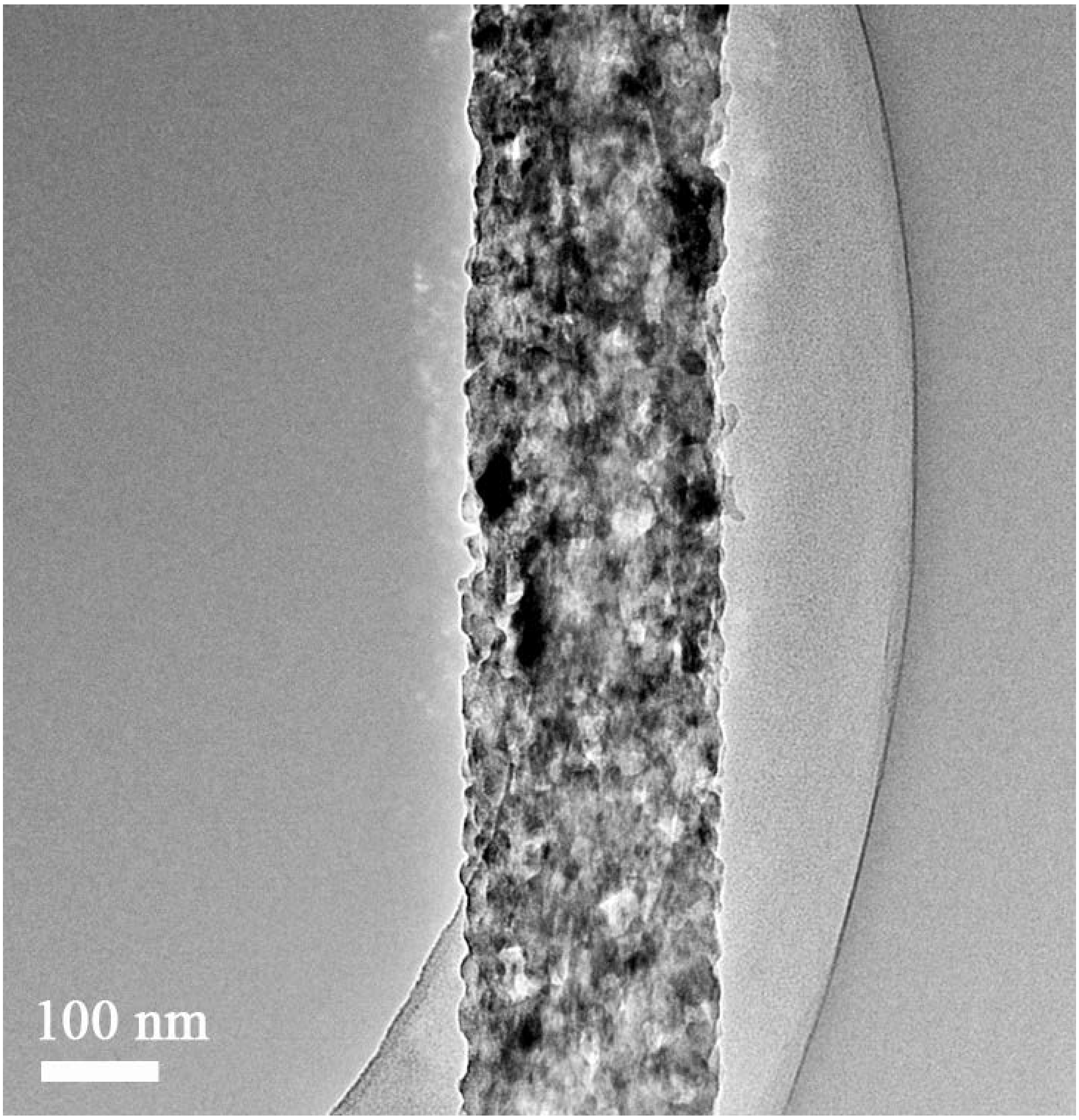

Supplementary Figure 8. Morphology of the pristine MgO nanowire. The pristine MgO nanowire fabricated through a hydrothermal method is porous polycrystalline.

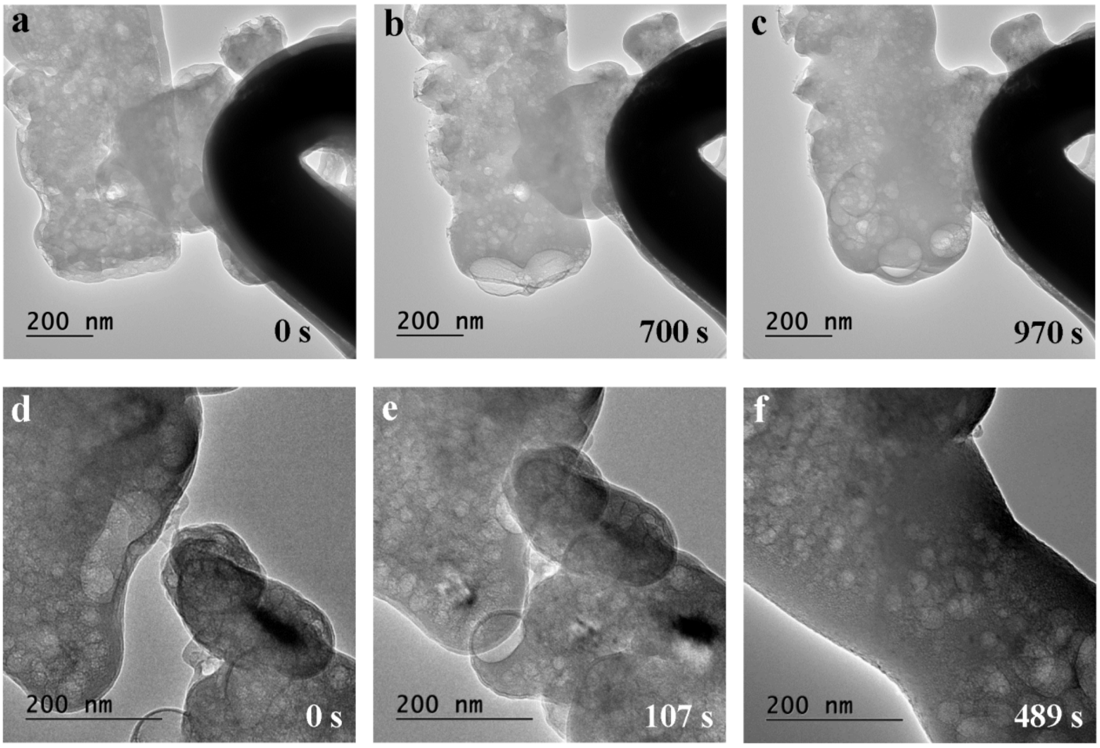

Supplementary Figure 9. The original junction interfaces disappeared after welding. a-c, The MgO was welded to a W tip in a side by side mode. **d-f**, Two MgO nanowires were welded together.

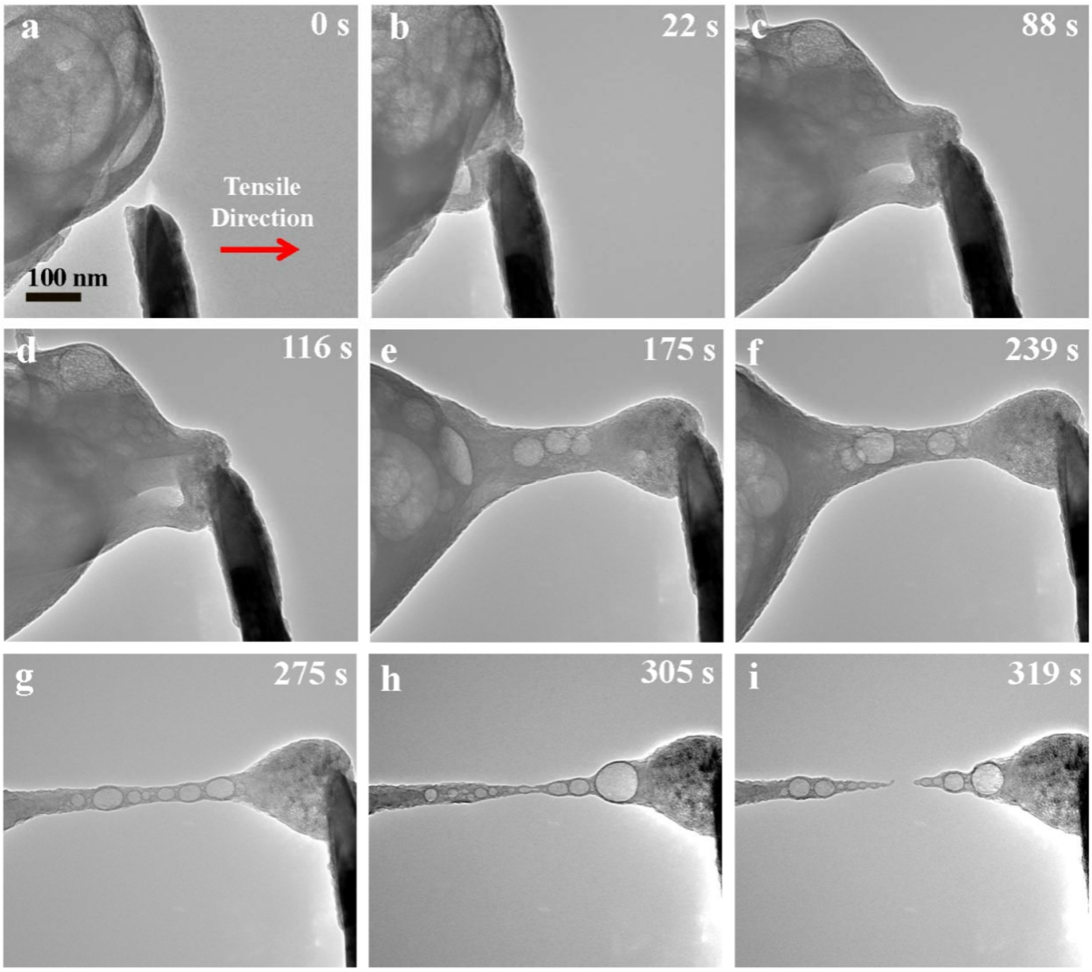

Supplementary Figure 10. A tensile test for the byproduct $MgCO_3$. a, The byproduct $MgCO_3$ obtained immediately after the carbonation of MgO. Red arrow in **(a)** indicates the pulling direction. **b-i**, The $MgCO_3$ displays a typical superplasticity phenomenon.

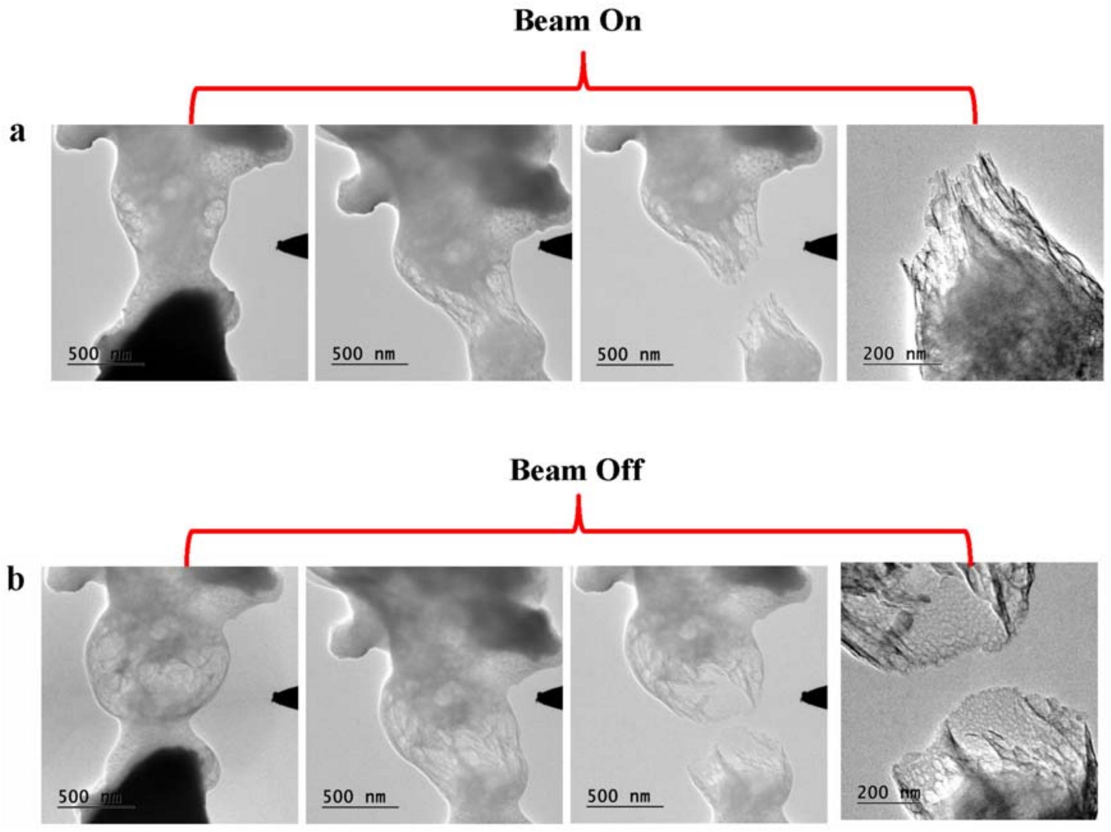

Supplementary Figure 11. Exploring the beam effect for the mechanical properties of the weld junction. Tensile tests of the weld junction under (**a**) Beam On, and (**b**) Beam Off conditions.

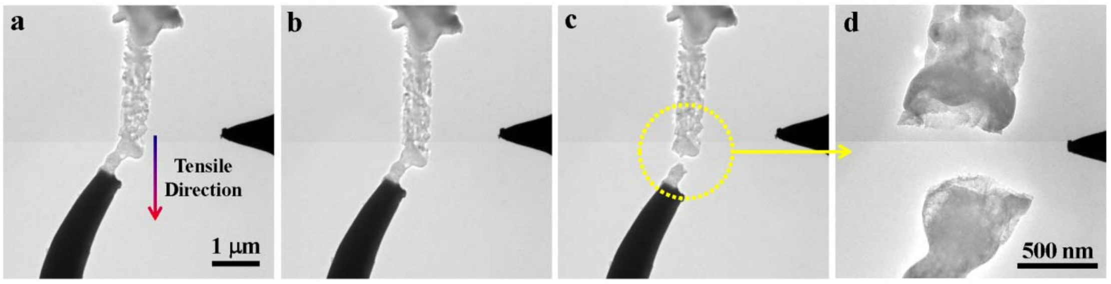

Supplementary Figure 12. Voids existing in the welding junction is destructive to the mechanical property of a welding. a-c, A tensile test for the MgO nanowire welded onto the W tip. Plenty of voids existed in the welded junction. The MgO nanowire broke on the welded junction. **d**, A magnified image of the fracture surface.

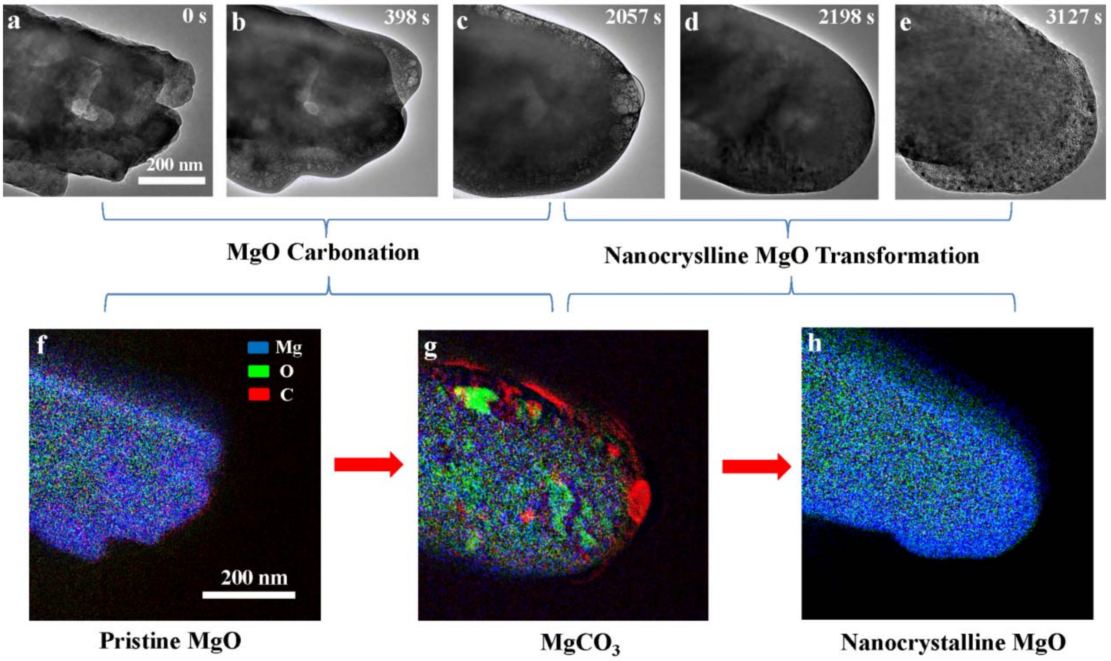

Supplementary Figure 13. A detailed transformation process of the pristine porous MgO to nanocrystalline MgO. a-e, The morphology evolution from the pristine porous MgO to a nanocrystalline MgO. **f-h**, The related energy filtered TEM (EFTEM) mappings of the pristine MgO, $MgCO_3$, and nanocrystalline MgO.

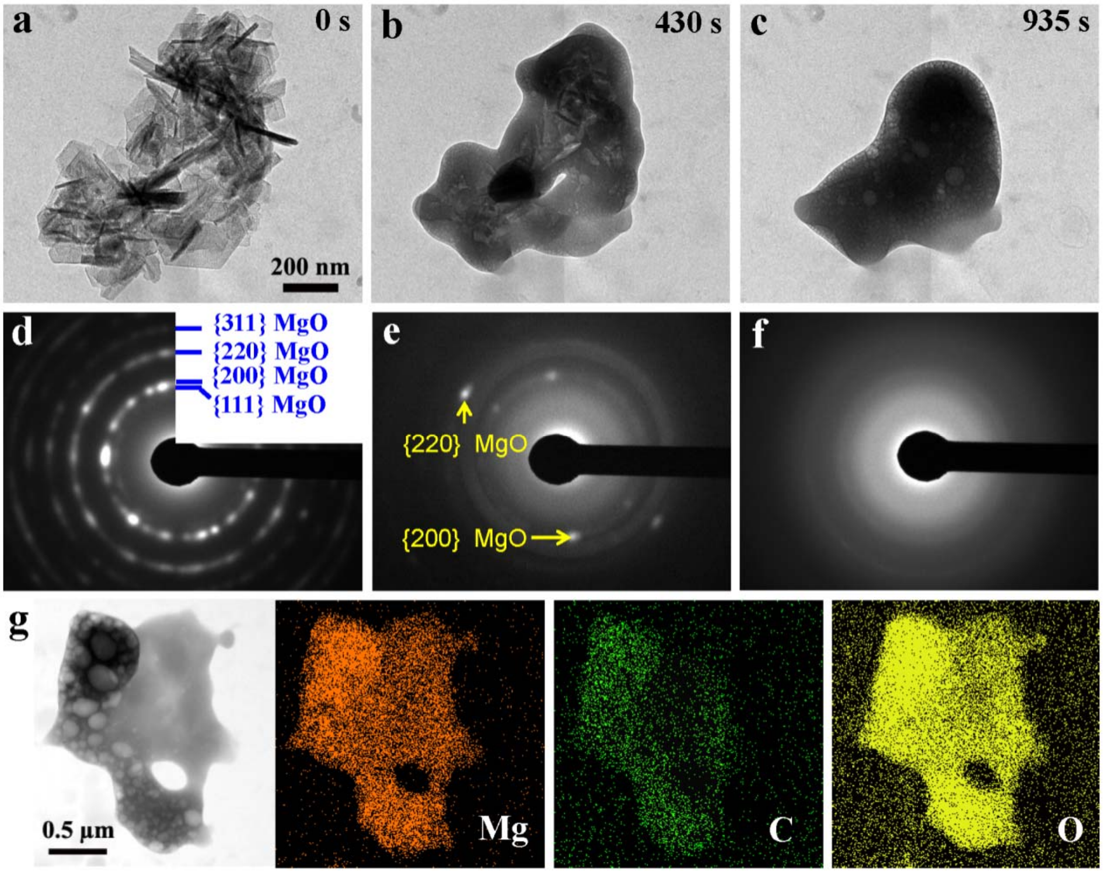

Supplementary Figure 14. Structure evolution of the MgO nanosheets under e-beam irradiation in a CO_2 environment. a-c, The morphology changes of the MgO nanosheets under e-beam irradiation in a CO_2 environment. **d-f**, EDPs corresponding to the MgO nanosheets in **a-c**, respectively. **g**, The STEM image of the byprodcut and its corresponding elemental mapping.

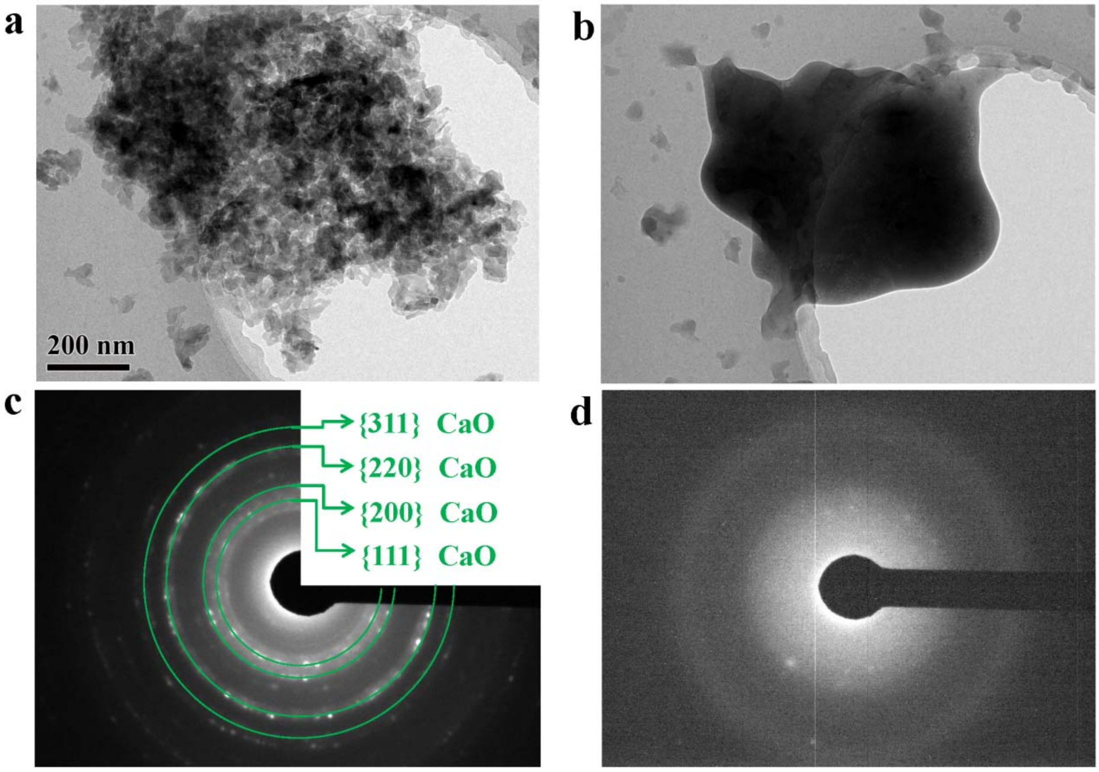

Supplementary Figure 15. The chemical absorption of CO_2 by using CaO nanoparticles under e-beam irradiation in a CO_2 environment. a,**c**, The TEM morphology and the corresponding EDP of the pristine CaO nanoparticles. **b**,**d**, The TEM morphology and the corresponding EDP after reacting with CO_2 under the e-beam irradiation.

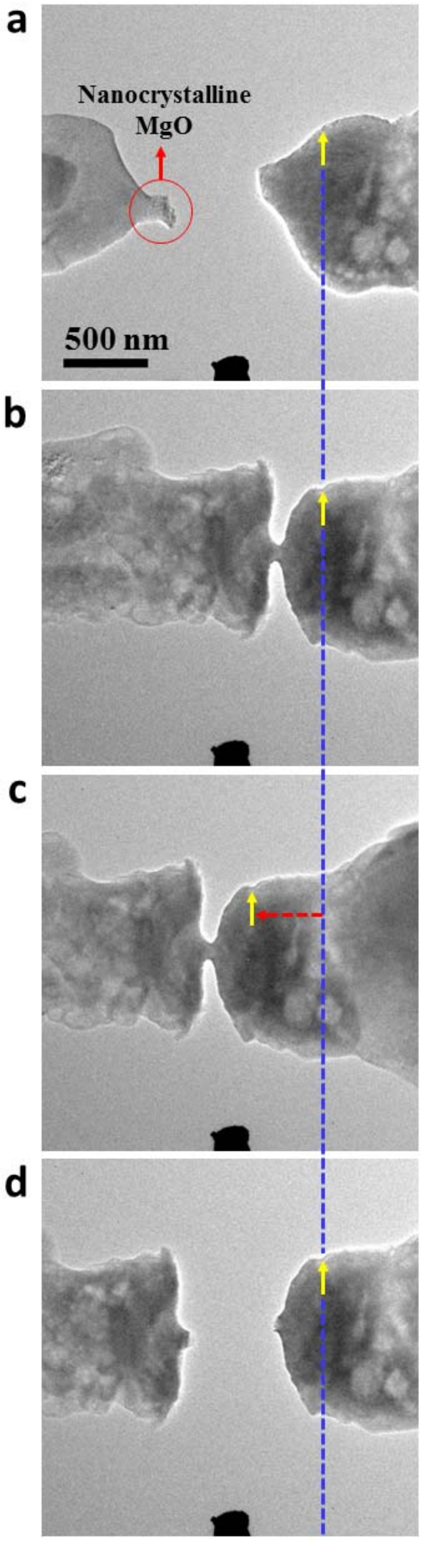

Supplementary Figure 16. In situ tensile tests of the weld junction comprised of the MgO nanocrystals. A MgO weld junction is created by the current ceramic welding technique (a, b), and then pulled until breakage (c, d).

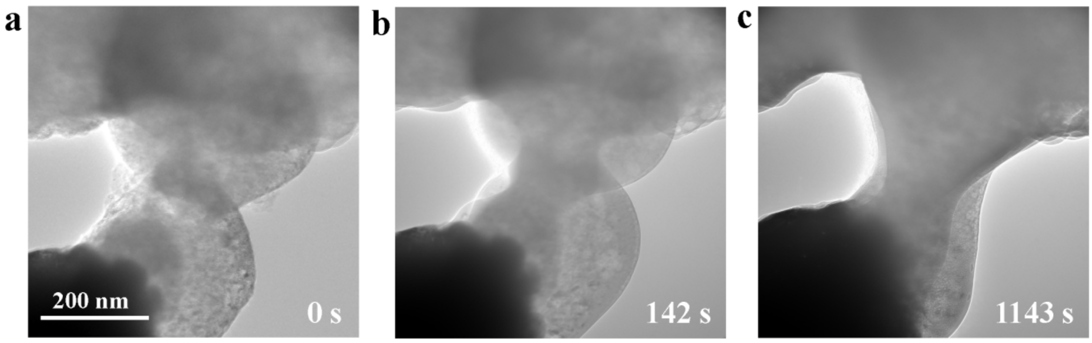

Supplementary Figure 17. After the tensile test, CO_2 gas is repumped into the ETEM chamber and e-beam irradiation started again to re-weld the fractured surfaces together again. a, The broken MgO surfaces are brought into close proximity. **b**, The welding starts. **c**, The welding is complete, and the fracture surfaces totally disappear.

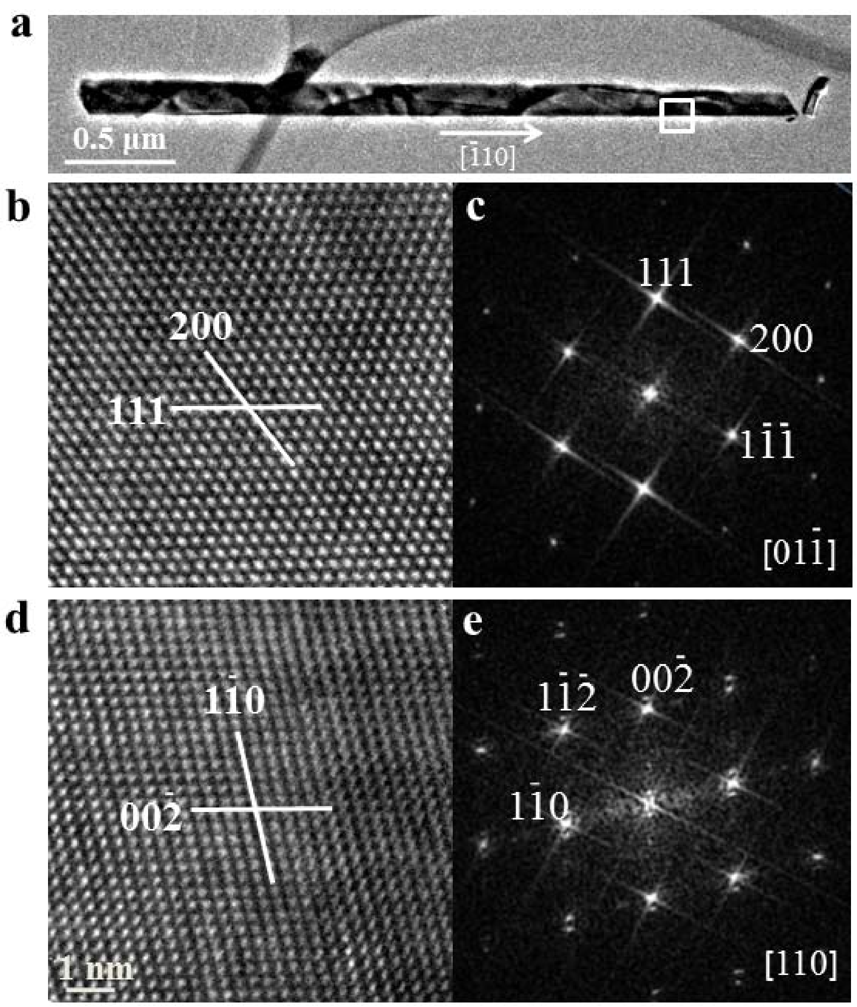

Supplementary Figure 18. The morphology and structure of a CuO nanowire. a, A TEM image of a CuO nanowire prepared by heating a copper grid at 500 °C for 4 h. **b, d**, High-resolution TEM images, showing the single crystal structure of a nanowire from two different zone axes. **c, e**, Fast Fourier transformation (FFT) patterns of the areas framed in **b, d,** respectively. These results indicate that the CuO nanowire is a single crystal.

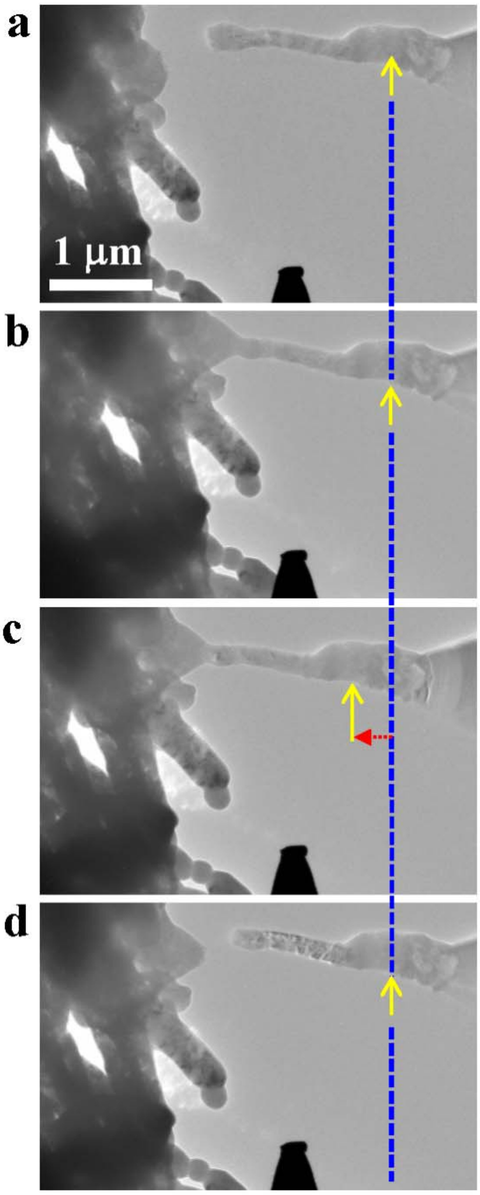

Supplementary Figure 19. In situ TEM tensile test of a CuO nanowire. a-d, The CuO nanowire was glued to a W tip on the left, and welded to the AFM cantilever on the right using the ceramic nanowelding technique reported in this paper. The AFM cantilever was displaced 381 nm until broken.

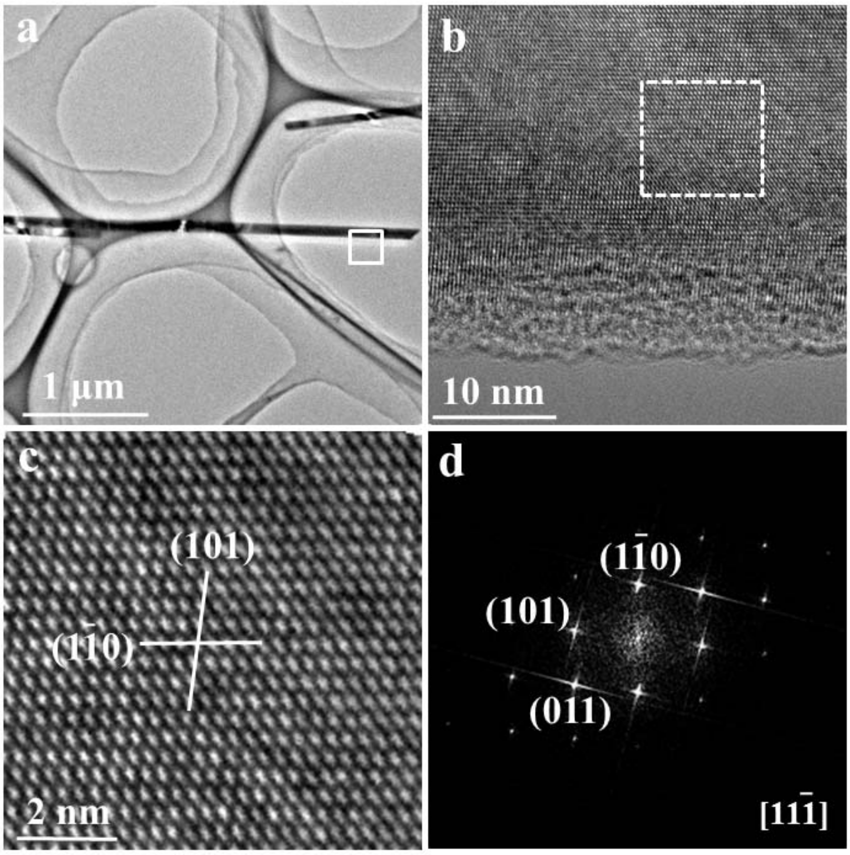

Supplementary Figure 20. The morphology and structure of a V_2O_5 nanowire. a, A TEM image of a V_2O_5 nanowire prepared by a hydrothermal method. **b**, A high-resolution TEM image obtained from the edge of the V_2O_5 nanowire. **c**, A high-resolution TEM image showing the single crystal structure of the nanowire. **d**, The FFT pattern of the area framed in **b**. These results confirm that V_2O_5 nanowire is a single crystal.

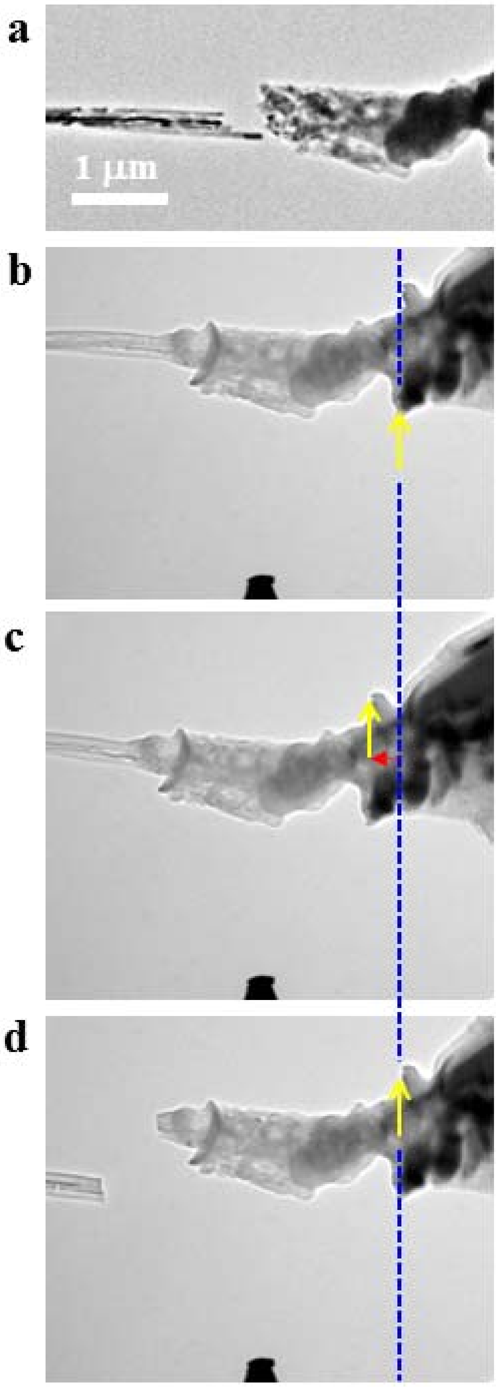

Supplementary Figure 21. In situ TEM tensile test of a V_2O_5 nanowire. a-d, The V_2O_5 nanowire was glued to a W tip on the left, and welded to the AFM cantilever on the right using the ceramic nanowelding technique reported in this paper. The AFM cantilever was displaced 303 nm until broken.

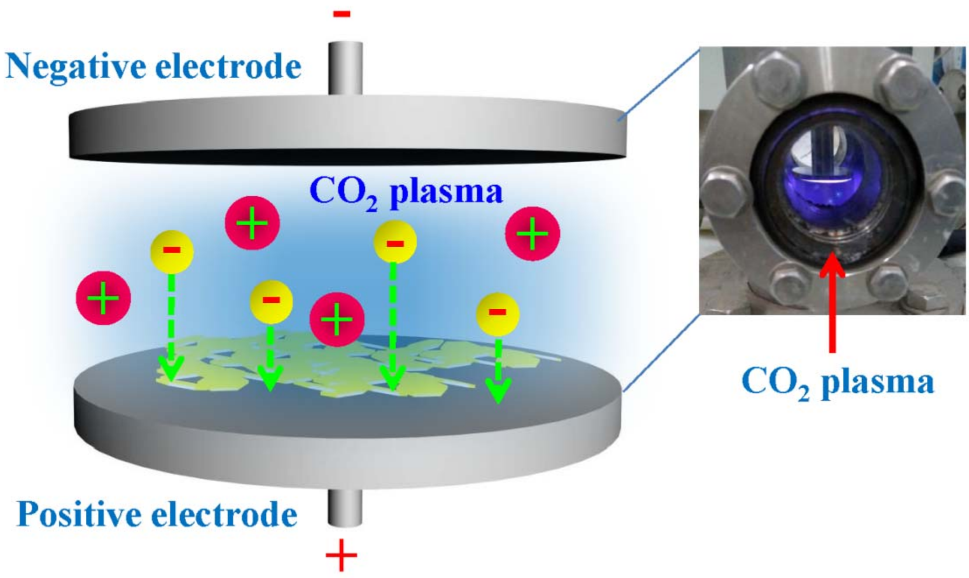

Supplementary Figure 22. The generation of CO_2 plasma. The schematic drawing and photograph of the plasma processing technology for macroscopic welding of ceramic materials. The macroscopic ceramic welding was achieved using this setup.

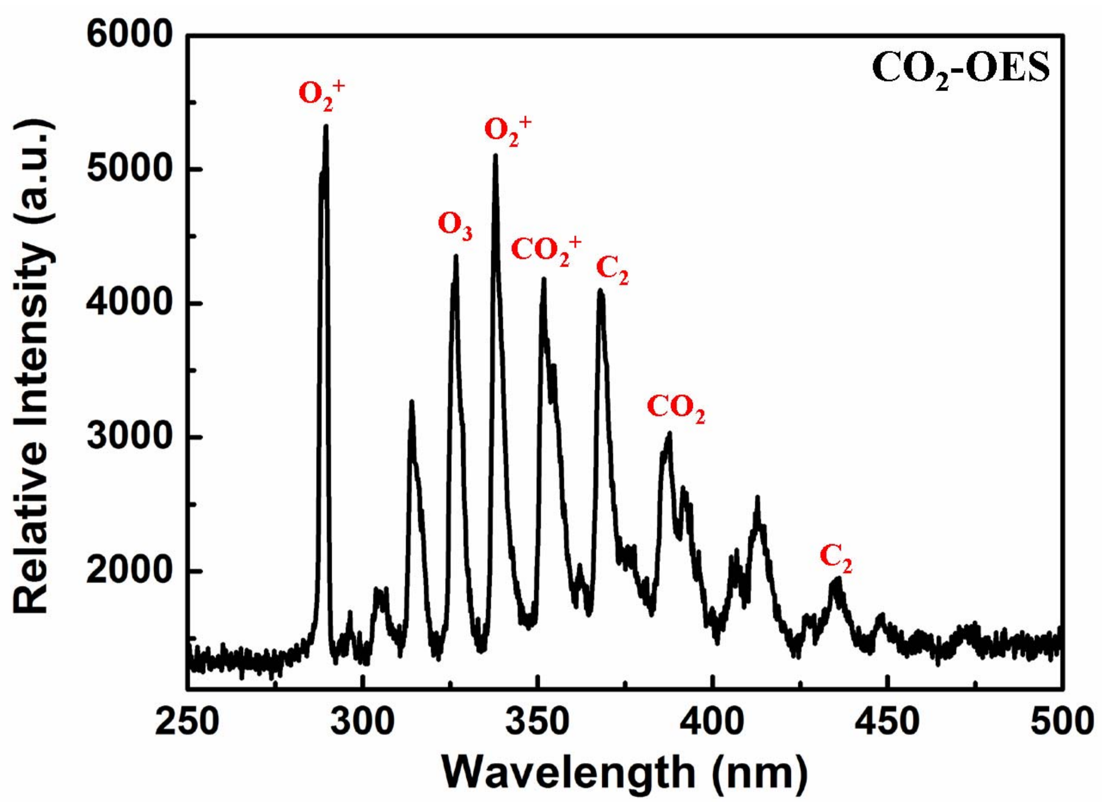

Supplementary Figure 23. The optical emission spectrum (OES) of the CO_2 under plasma. The CO_2 molecules are separated and activated under the plasma irradiation.

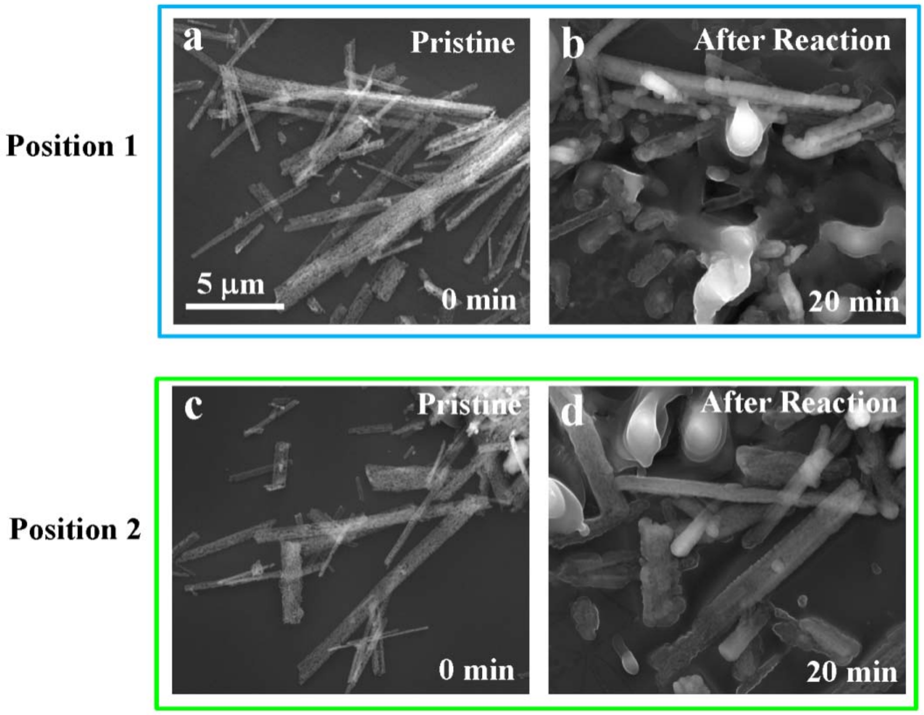

Supplementary Figure 24. The MgO deposited on the Si wafer before and after CO_2 plasma treatment. Two random positions are displayed here: **a**,**b**, Position 1, **c**,**d**, Position 2.

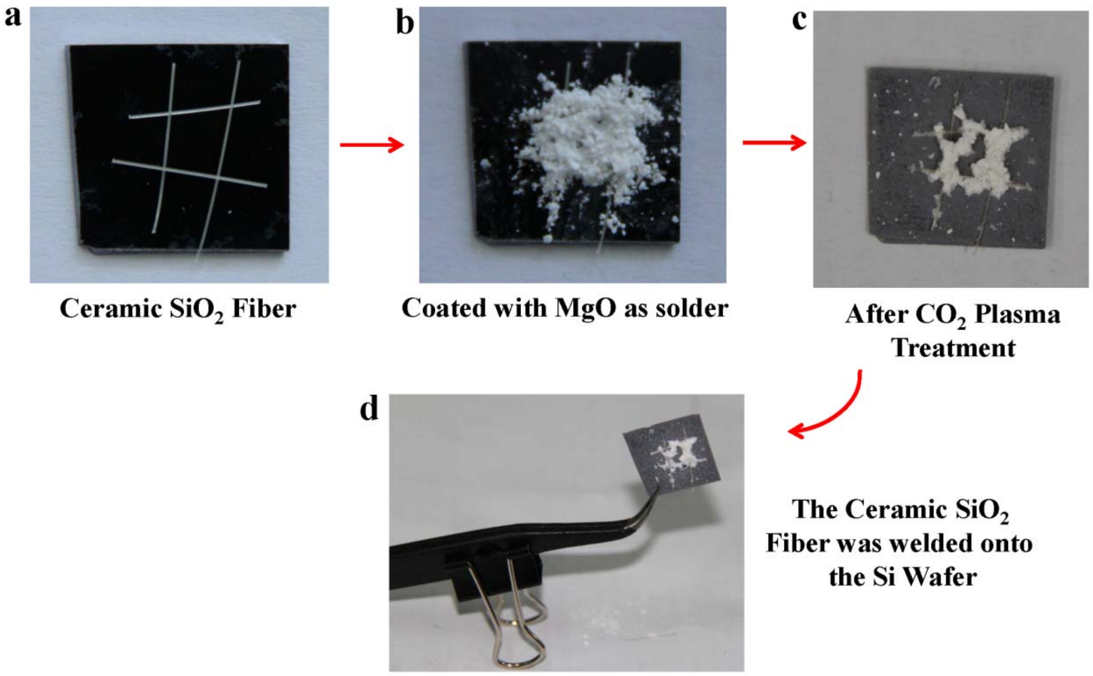

Supplementary Figure 25. A macroscopic ceramic glass fiber was welded onto the Si wafer by using the ceramic welding technique. a, Dropping some pristine SiO_2 fibers onto the Si wafer. **b**, Coating the SiO_2 fibers with MgO powder. **c**, After CO_2 plasma treatment, the glass fibers are successfully welded onto the Si wafer. **d**, Putting the welded sample in a vertical position, showing the SiO_2 fibers are welded onto the substrate.

Supplementary Methods

Supplementary Method 1. The fabrication of MgO nanowires

In a typical experiment, 29 g $MgSO_4 \cdot 7H_2O$ was mix with 30 mL deionized water by magnetic stirring for 10 min. 30 mL Ammonia solution (10 wt%) was slowly added into the mixture under stirring and continuing stirring for 20 min. The mixture was subsequently loaded into a Teflon-lined autoclave (100 mL capacity). The stainless steel autoclave was sealed and fixed into a homogeneous reactor and heated to 180 °C for 10 h. Thereafter, the autoclave was cooled to room temperature, the precipitates collected by filtration after being repeatedly washed with ethanol and deionized water, and dried in an oven at 60 °C. The dried powder was finally put into Muffle furnace and heated to 950 °C for 2 h and then cooled to room temperature.
