# Supplementary material for: Ceramic nanowelding
Source: Nat Commun. 2018 Jan 8;9:96. doi: 10.1038/s41467-017-02590-1 (PMC5758820; doi:10.1038/s41467-017-02590-1)
Supplement: Supplementary file 3 — Description of Additional Supplementary Files [file 41467_2017_2590_MOESM3_ESM.pdf]

Description of Additional Supplementary Files

File Name: Supplementary Movie 1
Description: An in situ TEM movie showing the MgO nanowire become fluidic and flow like a porous viscous liquid. The movie was recorded at 5 frames/second, and is played at 10× speed.

File Name: Supplementary Movie 2
Description: The nanowelding of the MgO nanowires was achieved three times in the ETEM chamber. The movie was recorded at 5 frames/second, and is played at 50× speed.

File Name: Supplementary Movie 3
Description: A tensile test for the $MgCO_3$ obtained immediately after reaction, showing the superplasticity of the $MgCO_3$. The movie was recorded at 5 frames/second, and is played at 50× speed.

File Name: Supplementary Movie 4
Description: A tensile test of a welding spot with plenty of voids existed in it, showing fracture occurred at a location where big voids were present. The movie was recorded at 5 frames/second, and is played at 40× speed

File Name: Supplementary Movie 5
Description: A detailed structure evolution of the nanowelding process. The movie was recorded at 5 frames/second, and is played at 20× speed.

File Name: Supplementary Movie 6
Description: The detailed generation process of nanocrystalline MgO. The movie was recorded at 5 frames/second, and is played at 100× speed.

File Name: Supplementary Movie 7
Description: A tensile test of a welding spot comprised of pure nanocrystalline MgO. The movie was recorded at 5 frames/second, and is played at 20× speed.

File Name: Supplementary Movie 8
Description: Several tensile experiments for the MgO, CuO, and V_2O_5 nanowires. The nanowires were fixed to the AFM cantilever by using the ceramic nanowelding technology. The movie was recorded at 5 frames/second, and is played at 100× speed.

File Name: Supplementary Movie 9
Description: The welding of ceramic SiO_2 fibers in macroscale by using MgO as the solder. The movie was recorded at 5 frames/second, and is played at 20× speed.
